# Prognosticators for Visual Outcome in Indirect Traumatic Optic Neuropathy: A Prospective Cohort Study

**DOI:** 10.7759/cureus.35344

**Published:** 2023-02-23

**Authors:** Sangeeta Gupta, Alka Tripathi, Gaurav Gupta

**Affiliations:** 1 Physiology, All India Institute of Medical Sciences, Gorakhpur, IND; 2 Ophthalmology, All India Institute of Medical Sciences, Gorakhpur, IND; 3 General Surgery, All India Institute of Medical Sciences, Gorakhpur, IND

**Keywords:** optic neuropathy, traumatic optic neuropathy, amplitude, latency, predictors, visual evoked potential, visual acuity, indirect traumatic optic neuropathy

## Abstract

Introduction

Traumatic optic neuropathy (TON), with indirect TON as its more prevalent form, is a dreadful cause of severe visual dysfunctions. The condition is known to have a contentious treatment plan and poor visual sequelae; hence, the assessment of prognostic signs becomes valuable. Prospective studies evaluating important predictors of visual recovery after traumatic optic nerve injury can particularly be helpful in a longitudinal observation. The possible roles of clinical variables need to be assessed. Absent visual evoked potential (VEP) records as a crucial finding associated with TON has reportedly valuable prognostic significance. This also needs to be explored. Hence, the study sought to determine the role of prognosticators in the visual outcome of the patients, with a focus on evaluating the role of VEPs in the severity and prognosis of indirect TON.

Methods

A prospective observational study involving 40 patients with indirect TON was conducted. Ocular, neuro-ophthalmological, radiological, and neurophysiological variables, including flash VEP, were investigated at their initial visit and followed up until the end of six months. Final visual acuity was the primary outcome variable studied. Paired t-test was used to perform the comparison between the flash VEP variables for normal and affected eyes at the initial visit. Pearson correlation coefficient was computed for obtaining the association of initial visual acuity and flash VEP variables with the outcome variable. Relative risk was calculated and analysed for the prognosticators in univariate analysis. Statistical significance was defined as p < 0.05.

Results

Statistically significant variations in mean P100 latency, N75-P100, and P100-N145 amplitudes compared between normal and affected eyes in the patients at the initial visit were obtained (p < 0.0001; paired t-test). Pearson correlation coefficient for initial visual acuity and flash VEP variable as independent variables and final visual acuity as the dependent variable were statistically significant (p < 0.05). The relative risks for prognosticators with a statistically significant range of confidence intervals were poor initial visual acuity, greater relative afferent pupillary defect (RAPD) grades, deranged flash VEP variables (absent VEP, reduction in amplitude ratio (>50%), and increased interocular latency differences), loss of consciousness during injury, age greater than 40 years, and lack of improvement after 48 hours of steroid treatment.

Conclusion

The identified negative prognosticators may be helpful in deciding the kind of therapeutic approach and predicting the visual outcome in patients with indirect TON.

## Introduction

Traumatic optic neuropathy (TON) is a rare yet catastrophic cause of substantial loss of vision. The rate of incidence varies in different countries; the United States has 0.5-5% of patients with closed head trauma and 0.7-2.5% with midfacial fractures [1,2]. A recent national epidemiological survey conducted in the United Kingdom reports a prevalence of TON as one in 1,000,000, with young males affected in their early 30s [3]. In India, over 10,000 patients with head trauma sustain indirect optic nerve injury and severe visual loss every year [4]. In research on rural Indian patients, closed globe injury was the most common type of ocular trauma, accounting for 80% of cases [5].

According to the mode of injury, the condition has been classified as direct or indirect. Indirect TON is more common, which results due to impact transmitted to the optic nerve without direct damage to the ocular tissues as found in closed globe trauma patients. The common site of injury is the intracanalicular segment of the optic nerve. Compression and disruption of the pial vessels within the canal limiting the vascular supply of the optic nerve has been implicated as a result of the insult [6-8].

Indirect TON is characterized by a normal fundus examination (mostly in posterior injuries, which are more common), normal magnetic resonance imaging (MRI) and computed tomography (CT) scan of the optic nerve and canal with reduced vision (variable loss of vision ranging from normal to no light perception), relative afferent pupillary defect (unilateral TON), variable visual field loss, and loss of colour vision. Orbital CT scans, however, may detect acute orbital haemorrhages and orbital wall fractures [1].

Management of patients with TON is controversial with no proven treatment guidelines. A meta-analysis by Cook et al. reports significant improvement in patients receiving large doses of steroids or optic canal decompression as compared to those with no treatment [9]. However, the data are yet to be supported by other similar studies [10].

Prognostic signs in the patients during the first visit might help in deciding the course of treatment (medical, surgical, or no treatment), and the final visual outcome in the patients has been found to be affected by them. Cook et al. (1996), in their meta-analysis, concluded that treatment with corticosteroids, extracranial decompression, or both is better than no treatment of TON [9]. In their comparative nonrandomized interventional study, Levin et al. in 1999, however, concluded that neither corticosteroids nor optic canal surgery should be considered the standard of care for patients with TON and the treatment should be decided on an individual patient basis [10]. Prognostic indicators hence can help in guiding the treatment plan when no standard protocol is suggested to be followed.

The prognosis for indirect TON has been found to be better than for direct TON, but a high level of clinical vigilance has been advised in these patients since the development of an optic nerve sheath haematoma subsequently can result in further delayed visual loss. This also necessitates a long-term follow-up of the patients.

Patients with no light perception at presentation have revealed limited or no visual improvement. Other poor prognostic factors include loss of consciousness, lack of visual recovery after 48 hours, and absence of visual evoked responses [11-15]. Visual evoked potential (VEP) tests have been found to be of great value in assessing the severity as well as the visual recovery potential of the patients. Assessment of optic nerve function by testing pupillary reactivity may be severely compromised or impossible because of tensely swollen eyelids, conjunctival oedema, and concussion of the ciliary muscle. Electrophysiological testing by VEP may help in optic nerve functions in such patients. Many studies emphasize the high predictive value of VEP in optic nerve injuries [16-19].

Both pattern reversal and flash VEP testing have been described as helpful in predicting visual acuity in the literature with a reduction of amplitude in the flash VEP being of particular importance [20,21]. The presence of the waveform as well as the amplitude of VEP has been demonstrated to be predictive of long-term outcomes. When the VEP is absent, recovery of vision is poor and unlikely. In unilateral cases, the normal side can act as a control and amplitude ratios then reflect the outcome, which has been found to be favourable when the VEP amplitude is within 50% of the normal side [19]. VEP has also been reported to be of diagnostic value in patients who do not remember the time of nerve damage, patients with unreliable pupillary responses, and patients with bilateral TON. Some other ocular and systemic signs have also been suggested to be important predictors of indirect TON. Age, associated ophthalmoplegia, presence of fractures of the orbital wall, and relative afferent pupillary defect (RAPD) have been studied and described as predictors and signs of visual recovery [11-13,21]. Cases with initially negative prognostic signs have been reported to show clear benefits from treatment versus observation alone. Hence, the assessment of prognostic indicators can have a significant contribution.

Previous studies that evaluated the risk factors and the predictors of the outcome have, in the majority, been retrospective ones in nature [4,11]. Prospective cohort studies (with less bias) to evaluate the important predictors of visual recovery after traumatic optic nerve injury involving long-term follow-ups are very sparse [22]. Hence, the present study is planned in an attempt to evaluate various risk factors and predictors of visual recovery in patients with an indirect TON in a longitudinal fashion involving regular follow-ups till the end of six months after the initial visit. This study aimed at investigating the role of prognosticators (ocular, systemic, neurophysiological, and radiological) in the final visual outcome of the patients by a prospective evaluation with emphasis on assessing the role of VEPs in the severity and prognosis of indirect TON.

## Materials and methods

A prospective, observational study was conducted involving 40 patients with indirect TON (attending the ophthalmology and surgery department) at a tertiary care centre from December 2020 to November 2022. Sample size calculation was done on the basis of significant parameters’ odds ratio from a previous similar study [11]. Approval for conducting the study was obtained from the Institutional Human Ethics Committee (IHEC), All India Institute of Medical Sciences, Gorakhpur, India (reference number: IHEC/AIIMS-GKP/BMR/22/2020). Informed written consent was obtained prior to conducting the study.

The diagnosis was made by the presence of decreased visual acuity after a history of head trauma associated with an RAPD and after the exclusion of other possible diagnoses by the ophthalmologist. Patients with a history of neuro-ophthalmological disorders before the history of trauma were excluded. Also, cases which report post-traumatic loss of vision without optic nerve lesions (traumatic cataract, retinal detachment, etc.) were excluded.

Patients underwent ocular, neuro-ophthalmological, radiological, and neurophysiological examinations at their initial visit and were followed up till the end of six months. The ocular and neuro-ophthalmological examination included visual acuity, visual field examination, associated ophthalmoplegia, fundus examination, and RAPD. Visual acuity was recorded by Snellen’s chart. A numeric scale of visual acuity was obtained by estimating the best corrected visual acuity (BCVA) and converting it into a logarithm of the minimum angle of resolution (logMAR) units. Anterior segment examination was performed by torchlight and slit lamp biomicroscopy and pupillary examination (direct, consensual, and swinging flashlight tests) was performed. RAPD was graded conventionally as 1+ to 4+ with 4+ as no change in pupillary size. Final visual acuity was the outcome variable studied. Recovery of vision was defined as any improvement by at least one decimal fraction of Snellen visual acuity confirmed at the last follow-up visit.

**Figure 1:**
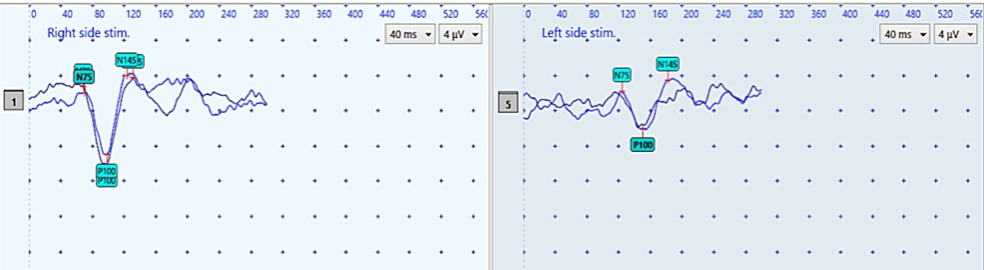
Flash VEP record of a patient with indirect TON (with a history of assault on the head) Diminished vision in the left eye, visual acuity in the right eye: 6/6 and left eye: HMFC. VEP findings demonstrate a 50% reduction in interocular amplitude ratio (left/right eye) with delayed P100 latency in the left eye (at the initial visit). VEP: visual evoked potential; TON: traumatic optic neuropathy; HMFC: hand movements close to the face.

Neurophysiological predictors included VEP examination. The methodology for the test employed was standardized as recommended by the American Clinical Neurophysiology Society guidelines [23]. Preparation of scalp skin was done prior to the electrode application. Standard surface electrodes (gold-plated cup electrodes) were placed according to the international 10/20 system of electrode placement, with an active electrode at Oz, a reference electrode at Cz, and a ground electrode at Fpz with Ten20 conductive paste and micropore tape. The inter-electrode impedance of less than 5 kohms was ensured before starting the test. The method of presentation of the stimuli was by means of goggles and the type of stimuli presented were flash stimuli (flash VEP). The flash goggles used red LED flash of 3 candela sm-2. The frequency of the stimulus was 1 Hz. The signals were amplified and filtered with a system bandpass filter of 2-100 Hz. A total of 200 responses were averaged. Recordings for normal as well as affected eyes were obtained. P100 latencies, N75-P100 amplitudes, and P100-N145 amplitudes were the VEP variables recorded and analysed. Interocular latency difference (affected eye - normal eye) and interocular amplitude ratios (affected eye/normal eye) were considered (Figure 1). Recordings were performed by a physiologist with expertise in electrophysiological tests.

An orbital CT was performed in all the patients to detect any orbital wall fracture. All the patients received high-dose steroid treatment within 72 hours of trauma [24].

All the baseline neuro-ophthalmological and radiological examinations were performed as soon as the mental state and physical condition of the patients were adequate. The examination was repeated after 48 hours of steroid treatment. Patients were then followed up one, three, and six months after the initial visit.

Statistical analysis

Mean ± standard deviation, numbers, and percentages were used to express numerical data. Paired t-test was assessed for quantitative variables (with normal distribution). The Pearson coefficient was used to find out the correlation between two normally distributed quantitative variables. Relative risk (RR) and confidence intervals (CIs) were calculated for the prognosticators (in univariate analysis) with visual acuity improvement (numeric scale) as the outcome variable. Data were analysed using SPSS software version 28.0.0 (IBM Corp., Armonk, NY). P-value < 0.05 was considered statistically significant.

## Results

Table 1 demonstrates the details of the patients included in the study. The mean age of the patients was 33 ± 8.76 years. Of the patients, 95% were male. Eye involvement was in the ratio of 6:5. Road traffic accident was the most common cause of trauma (70%). The mean duration of the patients’ initial visit after trauma was 2.8 (one to seven) days. Initial (baseline) visual acuity was variable (no light perception to 6/9) (Table 1).

**Table 1:** Demographic and clinical characteristics (at baseline; n = 40) SD: standard deviation; NLP: no light perception; LP: light perception; HM: hand movement; FC: finger counting; RAPD: relative afferent pupillary defect; VEP: visual evoked potential.

Characteristics	Total number (%)
Age (years), mean ± SD: 33 ± 8.76	>40 years: 12 (30%)
	≤40 years: 28 (70%)
Sex	
Male	38 (95%)
Female	02 (5%)
Eye involved	
Right	22 (55%)
Left	18 (45%)
Nature of injury	
Road traffic accident	28 (70%)
Fall	9 (22.5%)
Assault	3 (7.5%)
Baseline visual acuity	
NLP	2 (5%)
LP	3 (7.5%)
HM	3 (7.5%)
6/60 to FC	12 (30%)
6/24 to 6/36	16 (40%)
6/6 to 6/18	4 (10%)
CT scan finding - orbital wall fracture	
Present	11 (27.5%)
Absent	29 (72.5%)
RAPD	40 (100%)
Ophthalmoplegia	6 (15%)
3^rd^ nerve palsy	3
6^th^ nerve palsy	2
Total ophthalmoplegia	1
Flash VEP absent response or >50% reduction in interocular amplitude ratio	12 (30%) (absent VEP response in the affected eyes: 6; >50% reduction in interocular amplitude ratio: 6)
≤50 reductions in interocular amplitude ratio	28 (70%)
Loss of consciousness at the time of injury	
Present	15 (37.5%)
Absent	25 (62.5%)
Improvement after 48 hours of steroid treatment	
Present	18 (45%)
Absent	22 (55%)

**Figure 2:**
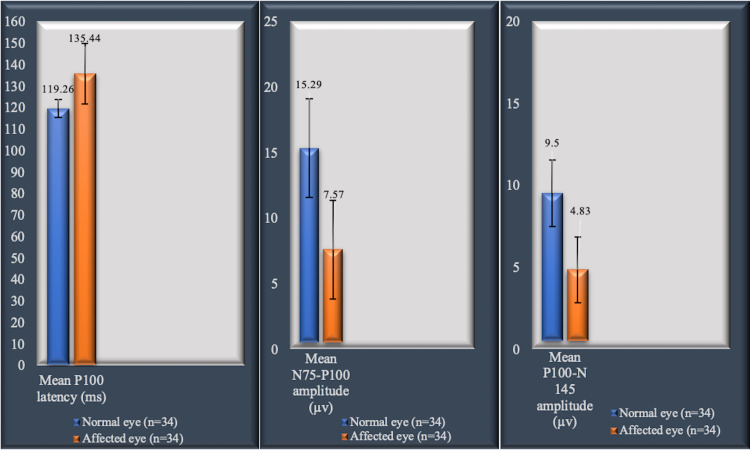
Comparison of VEP variables (mean P100 latency (ms), N75-P100 amplitude (µv), and P100-N145 amplitude (µv)) between normal eyes and affected eyes in patients with indirect TON at the initial visit (n = 34; six patients with absent VEP response) VEP: visual evoked potential; ms: milliseconds; µv: microvolts; TON: traumatic optic neuropathy.

The correlation of initial visual acuity with the final (numeric scale) was found to be statistically significant (r = 0.48; p < 0.05; Table 2). Similarly, a correlation study for the association of flash VEP variables with the outcome variable (final visual acuity) showed a statistically significant correlation for the interocular amplitude ratios as well as latency values (p < 0.05; Table 2). Relative risk calculation was performed for initial visual acuity classifying it into poor (<6/60) and better (≥6/60), and it was found to be 2.5 with statistical significance (Table 3). The presence of orbital wall fractures was found in 27.5% of patients, which was not found to have a significant increase in the risk of no improvement in visual acuity (RR: 1.4) (Table 3). Ophthalmoplegia found in 15% of the patients with a relative risk of 1.42 was not found to have statistical significance (Table 3). Flash VEP variables compared between normal and affected eyes in the patients at the initial visit showed statistically significant variations in the mean P100 latency (119.26 ± 4.16 vs. 135.44 ± 14.09) and both the amplitudes studied, i.e., N75-P100 and P100-N145 (15.29 ± 3.76 vs. 7.57 ± 3.75 and 9.5 ± 2.02 vs. 4.68 ± 2.0, respectively) (p < 0.0001; paired t-test) (Figure 2).

**Table 2:** Correlation of initial visual acuity and flash VEP variables (independent quantitative variables) with final visual acuity (dependent quantitative variable) VEP: visual evoked potential.

Variable	Correlation coefficient value (r)	P-value
Visual acuity (numeric scale) (n = 40)	0.48	0.002 (p < 0.05)
Flash VEP variables (n = 34)		
Interocular P100 latency difference	-0.45	0.007 (p < 0.05)
Interocular amplitude ratio (N75-P100 amplitude)	0.6	0.0001 (p < 0.05)
Interocular amplitude ratio (P100-N145 amplitude)	0.5	0.0026 (p < 0.05)

**Table 3:** Relative risk assessment for the prognosticators (univariate analysis) with visual acuity improvement (numeric scale)* as the outcome variable (n = 40) * LogMAR units (logarithm of the minimum angle of resolution). RAPD: relative afferent pupillary defect; VEP: visual evoked potential.

Clinical variable (number of cases)	No. of cases with no improvement	No. of cases with improvement	Relative risk (RR)	95% confidence interval	P-value
Initial visual acuity					
<6/60 (20)	15	5	2.5	1.22 to 5.11	0.0121
≥6/60 (20)	6	14			
RAPD grade					
High grade (+3 and +4) (12)	10	2	1.67	1.0 to 2.61	0.0256
Low grade (+1 and +2) (28)	14	14			
Ophthalmoplegia					
Present (6)	5	1	1.42	0.89 to 2.23	0.1336
Absent (34)	20	14			
CT scan findings					
Fracture (orbital wall)					
Present (11)	8	3	1.40	0.85 to 2.33	0.18
Absent (29)	15	14			
Flash VEP variables					
Interocular latency P100 latency difference (n = 34)					
Present (17)	13	4	2.6	1.19 to 5.69	0.017
Absent (17)	5	12			
Absent response or >50% reduction in interocular amplitude ratio (12)	10	2	2.33	1.33 to 4.07	0.0029
≤50 reductions in interocular amplitude ratio (28)	10	18			
Loss of consciousness at the time of injury					
Present (15)	12	3	1.81	1.09 to 3.02	0.0215
Absent (25)	11	14			
Improvement after 48 hours of steroid treatment					
Absent (22)	15	7	12.27	1.78 to 84.21	0.0107
Present (18)	1	17			
Age					
>40 years (12)	9	3	1.75	1.02 to 2.99	0.041
≤40 years (28)	12	16			

The relative risks calculated for absent VEP response and decreased interocular amplitude ratio (RR = 2.33), and interocular latency difference were found to be statistically significant (RR = 2.6; Table 3). High RAPD grades were also found to have a significant risk of no improvement (RR = 1.67; Table 3). Other variables associated with no recovery were loss of consciousness at the time of injury, no improvement after 48 hours of steroid treatment, and age > 40 years (Table 3).

## Discussion

Indirect optic nerve injury in patients with head trauma has been associated with severe visual loss. In a condition with a controversial treatment plan and poor visual sequelae, the assessment of prognostic signs proves to be valuable. The present study prospectively evaluated important predictors of visual recovery after traumatic optic nerve injury with a long-term follow-up. The role of reportedly important variables was assessed. Absent VEP records and flash VEP amplitudes of less than 50% of the normal eye, which have been found to be crucial in anticipating poor visual outcomes in unilateral TON patients, were explored. Shearing of nerve fibres, interruption of blood supply, or secondary oedema and haemorrhage following shearing or transection of the optic nerve vessels have been implicated in indirect injury to the optic nerve following head trauma [1,8,17].

In our study, young male patients were found with greater percentages of involvement, which is in concordance with the previous findings in indirect TON [4,25]. Among the demographic variables, age (>40 years) was found to be associated with poor visual outcomes. Age-related axonal lipoperoxidation and membrane hydrolysis occurring after the trauma have been speculated to be involved in impaired recovery [11,26,27]. Regarding the nature of the injury, road traffic accident was the most common cause supported by other similar studies [28,29]. Involvement of the right eye in a little greater proportion, as found in the current study, was also found in previous studies [17]. However, the side of involvement has been variable in different studies [28,29]. A positive correlation of the baseline/initial visual acuity was found with that recorded at the end of the follow-up. Also, a significantly increased risk of poor outcome was found to be present as obtained by relative risk for the same (RR = 2.5) (Tables 2, 3). This finding strengthens the previous reports regarding the role of initial visual acuity in predicting the recovery in TON [17,30]. Another important variable found to be valuable in anticipating the recovery was RAPD grades. The present study obtained significantly increased risk associated with high RAPD grades (RR = 1.67; Table 3). This also conforms to the previous similar study by Alford et al. (2001) [31].

Flash VEP has been reported to have high predictive value in TON in recent studies [17-19,25]. The present study also analysed the VEP variables to assess their role as a prognosticator. Statistically significant variation in all the flash VEP variables (mean P100 latency, N75-P100, and P100-N145 amplitudes) compared between normal and affected eyes in the patients at the initial visit was obtained (p < 0.0001; paired t-test). Also, a negative correlation of interocular amplitude ratio and a positive correlation of interocular latency difference with the impairment in outcome variable were characteristically found. Holmes et al. (2004) emphasized that an amplitude ratio of <0.5 was associated with poor recovery [19]. In the present study, the relative risk for the absent VEP and decreased interocular amplitude ratios along with interocular latency difference were found to be statistically significant (Table 3). VEPs have been suggested as better tools for quantifying the functional integrity of the optic pathways than scanning techniques. Their valuable role in assessing visual functions has been well-stated following trauma. It is not unusual that compression of optic pathways immediately after severe trauma results in no recordable VEPs. They may become recordable days later when inflammation subsides [32].

There were few other important variables found to contribute to predicting the visual recovery in the present study like loss of consciousness during injury and no recovery after 48 hours of steroid treatment. The above risk factors were found to be associated with statistical significance (p < 0.05; Table 3). The presence of orbital fractures and associated ophthalmoplegia in our study, however, were not found to be significant predictors of visual loss, which is in concordance with the studies by Tabatabaei et al. (2011) and Lessell (1989) [17,33].

Limitations of the study

The study could not include optical coherence tomography, which is a non-invasive method of evaluating the relationship between retinal layer thickness and visual functions in patients with TON. Some advanced neuroimaging techniques such as diffusion-weighted imaging have been evaluated for optic nerve hyperintensity in TON, reporting a reduction in various diffusion tension imaging (DTI) parameters [34]. DTI could have been performed in the patients as imaging markers. The study demonstrates the crucial predictive role of VEP in TON, yet it also has the limitation of not being usually performed at the bedside for patients with multiple injuries. It has also been stated that the presence of concurrent brain injuries may sometimes mimic the findings of optic nerve damage [35]. Also, multivariate analysis, including additional outcome variables, could have been included in the study.

## Conclusions

Poor initial visual acuity, higher RAPD grades, absence of VEP, reduction in VEP amplitude ratio (>50%), increased interocular P100 latency differences, loss of consciousness during injury, age greater than 40 years, and lack of improvement following 48 hours of steroid treatment were the negative prognosticators found in the patients with an indirect TON in the current study. As there is no generalized agreement on which treatment is best for TON, the assessment of prognostic indicators can have important implications for the treatment plan in TON.
